# The Importance of Learning Skills as Perceived by Adolescents in a Hospital School in Romania vs. Their Non-Hospitalized Peers

**DOI:** 10.5334/cie.260

**Published:** 2026-04-01

**Authors:** Maria-Magdalena Jianu, Elisabeta Niță, Mihai Benchea

**Affiliations:** 1 Hospital School, Bucharest, Romania

**Keywords:** Hospital school, skills, learning, adolescents, lifelong learning

## Abstract

The purpose of this cross-sectional survey was to evaluate the perceived importance of key lifelong learning skills among adolescents with a history of oncology hospitalization compared to peers without such experience and to identify skills they perceived as underdeveloped. Competencies were categorized following the framework of the Council of the European Union (Jurnalul Oficial al Uniunii Europene, 2018). Hospitalized adolescents were found to more frequently prioritize STEM (23.7%), multilingual (15.8%), and digital skills (13.2%). Non-hospitalized peers emphasized entrepreneurial (21.1%) and STEM skills (15.7%). A higher proportion of non-hospitalized adolescents (31.0%) were unsure about important skills compared to hospitalized peers (7.9%). Regarding underdeveloped skills, hospitalized adolescents highlighted literacy (18.4%) and STEM (15.8%), while non-hospitalized peers reported STEM (19.7%) and entrepreneurial skills (12.7%). These findings suggest that hospitalized adolescents may demonstrate clearer awareness of essential competencies, whereas non-hospitalized peers more often express uncertainty. These exploratory results may point to the potential role of hospital schools in mitigating educational disruptions, supporting continuity of learning, and helping guide adolescents toward developing lifelong competencies aligned with European educational priorities.

## Introduction

Education and health are fundamental and interconnected rights of children and adolescents. While healthcare institutions aim to safeguard the right to health, hospital schools seek to support continuity of learning during medical treatment (Medical Association, 2013). For young people diagnosed with cancer, hospitalization is a traumatic event that can disrupt education and delay the acquisition of key competencies (Benigno & Fante, 2020; Delloso et al., 2021; Lum et al., 2017). In the European context, the Recommendation of the Council of the European Union (Jurnalul Oficial al Uniunii Europene, 2018) established eight key competencies for lifelong learning, emphasizing their role in preparing young people for social participation, employability, and personal fulfillment. International research shows that hospital schools may play a compensatory role in achieving these goals, reducing inequalities and helping students reintegrate into mainstream classrooms (Caggiano et al., 2021; Hen & Gilan-Shochat, 2022).

Building on this framework, the present study explored how hospitalized adolescents in Romania perceive the importance of these competencies compared to their non-hospitalized peers, and examined which skills they consider underdeveloped, with implications of their responses for aligning hospital education in Romania with European lifelong learning priorities.

## Methods

In May 2024, a structured questionnaire was administered to 109 adolescents: 38 with a history of oncology hospitalization and 71 without such experience. Participants were recruited from a hospital school program in Bucharest, Romania, and from mainstream schools in the same geographic area. All adolescents were enrolled in lower- or upper-secondary education. Parental consent and adolescent assent were obtained prior to participation. Participants’ ages ranged from 12 to 18 years old (*M* = 15.2 years), with 65 females and 44 males. The hospitalized group comprised students undergoing oncology treatment during their hospital stay; all of them received instruction through the hospital school program.

The questionnaire included two closed-ended questions: (1) *Which lifelong learning skills do you consider most important?* and (2) *Which lifelong learning skills do you feel remain underdeveloped?* Questionnaire items were predefined and categorized according to the eight key competencies established by the Council of the European Union (Jurnalul Oficial al Uniunii Europene, 2018): literacy, multilingualism, STEM (mathematics, science, technology, and engineering), digital skills, personal/social/learning-to-learn, civic responsibility, entrepreneurial skills, and cultural awareness. An additional option, “I do not know,” was included and treated as a separate valid category.

Directly adapted from the European Council framework (Jurnalul Oficial al Uniunii Europene, 2018), the definitions were reformulated as simple closed-ended questions to suit the participants’ age group. Participants received brief written definitions for each competency category to ensure clarity and reduce misinterpretation. Responses were entered independently by participants on line via a secure link and then coded by the researchers directly into the corresponding competency category. Descriptive statistics (frequencies and percentages) were calculated for each item. Group differences between hospitalized and non-hospitalized adolescents were assessed using nine separate 2 × 2 chi-square tests or Fisher’s exact tests when expected cell counts were below 5. All analyses were conducted with Epi Info™ version 7.2.5.0 (Centers for Disease Control and Prevention [CDC], 2023).

## Results

As illustrated in Table 1, hospitalized adolescents most frequently reported STEM (23.7%), multilingualism (15.8%), and digital skills (13.2%) as important lifelong learning competencies. By comparison, their non-hospitalized peers prioritized entrepreneurial skills (21.1%) and STEM (15.5%). Notably, 31.0% of non-hospitalized adolescents selected “I do not know” compared with only 7.9% of hospitalized peers.

Chi-square and Fisher’s exact tests were conducted separately for each competency. Results showed a statistically significant group difference only for the “I do not know” category, where non-hospitalized adolescents more frequently expressed uncertainty, χ^2^(1, *N* = 109) = 8.11, *p* = .004. No other differences reached statistical significance; however, non-significant trends emerged for literacy and entrepreneurial competencies.

**Table 1:** Perceived Important Skills of Adolescents by Group.


SKILL	HOSPITALIZED (*n* = 38)	NON-HOSPITALIZED (*n* = 71)

Literacy	4 (10.5%)	2 (2.8%)

Multilingual	6 (15.8%)	6 (8.5%)

STEM	9 (23.7%)	11 (15.5%)

Digital	5 (13.2%)	4 (5.6%)

Personal/social/learning	5 (13.2%)	6 (8.5%)

Civic	0 (0.0%)	2 (2.8%)

Entrepreneurial	4 (10.5%)	15 (21.1%)

Cultural	2 (5.3%)	4 (5.6%)

“I do not know”	3 (7.9%)	22 (31.0%)


*Note*. Separate chi-square or Fisher’s exact tests were conducted for each skill. A significant difference was found for “I do not know,” χ^2^(1, *N* = 109) = 8.11, *p* = .004, with non-hospitalized adolescents reporting uncertainty more frequently. No other comparisons were statistically significant. Separate chi-square or Fisher’s exact tests were conducted for each skill:
Literacy: χ^2^(1, *N* = 109) = 2.83, *p* = .093 (Fisher *p* = .180) Multilingual: χ^2^(1, *N* = 109) = 1.36, *p* = .243 (Fisher *p* = .336) STEM: χ^2^(1, *N* = 109) = 1.11, *p* = .292 Digital: χ^2^(1, *N* = 109) = 1.85, *p* = .174 (Fisher *p* = .272) Personal/social/learning: χ^2^(1, *N* = 109) = 0.60, *p* = .437 (Fisher *p* = .510) Civic: Fisher’s exact *p* = 1.000 (expected counts < 5) Entrepreneurial: χ^2^(1, *N* = 109) = 2.02, *p* = .155 (Fisher *p* = .190) Cultural: χ^2^(1, *N* = 109) = 0.01, *p* = .943 (Fisher *p* = 1.000) “I do not know”: χ^2^(1, *N* = 109) = 8.11, *p* = .004 → significant

Regarding underdeveloped skills, hospitalized adolescents reported literacy (18.4%), STEM (15.8%), and digital skills (13.2%) as the most challenging. Non-hospitalized peers indicated STEM (19.7%) and entrepreneurial skills (12.7%) as underdeveloped (see Table 2). Separate chi-square and Fisher’s exact tests did not result in statistically significant group differences. However, two comparisons approached statistical significance—literacy, χ^2^(1, *N* = 109) = 3.27, *p* = .071, and “I do not know,” χ^2^(1, *N* = 109) = 3.41, *p* = .065)—suggesting that hospitalized adolescents may perceive greater difficulties in literacy, whereas non-hospitalized peers more often reported uncertainty.

**Table 2:** Skills Reported as Underdeveloped by Adolescents by Group.


SKILL	HOSPITALIZED (*n* = 38)	NON-HOSPITALIZED (*n* = 71)

Literacy	7 (18.4%)	5 (7.0%)

Multilingual	3 (7.9%)	8 (11.3%)

STEM	6 (15.8%)	14 (19.7%)

Digital	5 (13.2%)	4 (5.6%)

Personal/social/learning	5 (13.2%)	9 (12.7%)

Civic	1 (2.6%)	1 (1.4%)

Entrepreneurial	4 (10.5%)	9 (12.7%)

Cultural	3 (7.9%)	3 (4.2%)

“I do not know”	4 (10.5%)	18 (25.4%)


*Note*. Separate chi-square or Fisher’s exact tests were conducted for each skill. No statistically significant differences were found. Literacy, χ^2^(1, *N* = 109) = 3.27, *p* = .071, and “I do not know,” χ^2^(1, *N* = 109) = 3.41, *p* = .065, approached significance. Separate chi-square or Fisher’s exact tests were conducted for each skill:
Literacy: χ^2^(1, *N* = 109) = 3.27, *p* = .071 (Fisher *p* = .106) → marginal Multilingual: χ^2^(1, *N* = 109) = 0.31, *p* = .577 (Fisher *p* = .744) STEM: χ^2^(1, *N* = 109) = 0.26, *p* = .614 Digital: χ^2^(1, *N* = 109) = 1.85, *p* = .174 (Fisher *p* = .272) Personal/social/learning: χ^2^(1, *N* = 109) = 0.01, *p* = .943 (Fisher *p* = 1.000) Civic: Fisher’s exact *p* = 1.000 (expected counts < 5) Entrepreneurial: χ^2^(1, *N* = 109) = 0.09, *p* = .766 (Fisher *p* = .772) Cultural: χ^2^(1, *N* = 109) = 0.36, *p* = .550 (Fisher *p* = .665) “I do not know”: χ^2^(1, *N* = 109) = 3.41, *p* = .065 (Fisher *p* = .082) → marginal

## Discussion

The results of this exploratory study suggest potential differences in perceptions between hospitalized and non-hospitalized adolescents, although only one category reached statistical significance. Hospitalized adolescents appeared to demonstrate clearer awareness of essential skills, which might reflect their experiences of vulnerability and resilience, while non-hospitalized peers more often expressed uncertainty, possibly indicating gaps in educational guidance.

Perceptions of underdeveloped skills were largely similar across the two groups. Hospitalized adolescents were more likely to indicate challenges in literacy and digital skills, whereas non-hospitalized peers emphasized STEM and entrepreneurial competences. These patterns are broadly consistent with previous research showing that health-related school interruptions increase risks of academic delay and reduced self-confidence (National Children’s Cancer Society, 2022; Schultz et al., 2007).

The findings resonate with European policy directions, which stress equity in education and the importance of targeted support for vulnerable learners. According to Plage et al. (2022), for example, participation in education for children with chronic illnesses should be analyzed through a life-course perspective, recognizing the long-term implications of interrupted schooling. Similarly, Lewandowska (2022) emphasized that meeting students’ psychosocial needs is inseparable from academic support, underscoring the need for hospital schools to integrate both dimensions.

From a practical perspective, these preliminary results suggest that hospital teachers could benefit from continuous professional development focused on digital pedagogy, individualized curriculum design, and collaboration with healthcare staff. Policy frameworks at the national level may consider integrating hospital schools more systematically into mainstream education planning, with the aim of aligning them with European educational standards.

Finally, the study highlights the need for further research with larger samples to confirm these exploratory findings. Parents of children with cancer face multiple needs, particularly psychological, emotional, and informational, which are not sufficiently addressed by the current health system (Lewandowska, 2022). Addressing both academic and psychosocial challenges is crucial, as emphasized in recent studies (Haley et al., 2024; Pini et al., 2013; Tine & Vincent, 2020). These findings may point to the potential value of collaborative educational planning from the time of diagnosis, integrating both academic and non-academic dimensions.

## Conclusions

Hospital schools may play an important role in supporting continuity of education for adolescents undergoing cancer treatment, potentially helping to prevent skill loss and promote reintegration into mainstream classrooms.

The findings of the current study emphasize (a) the perceived importance of lifelong learning skills in both hospitalized and non-hospitalized adolescents; (b) the need for continuous guidance in helping students align their learning with EU 2018 competencies; and (c) the potential role of hospital teachers in adapting curricula to overcome medical and social barriers.

Although based on a small sample size and conducted within a limited timeframe, the findings offer preliminary insights that may support the development of specialized educational strategies in hospital schools. Strengthening collaboration between healthcare and education systems could be a key step in promoting equitable access to lifelong learning, and future research should explore long-term educational outcomes for hospitalized adolescents with cancer. Finally, by acknowledging the perspectives of hospitalized adolescents, this exploratory study highlights the importance of integrating hospital schools into national education policies, with the goal of fostering equity, resilience, and lifelong learning opportunities for young people with cancer.
